# Design and Analysis of a Bionic Pressing Roller Based on the Structural Characteristics of Pangolin Scales

**DOI:** 10.3390/biomimetics11010050

**Published:** 2026-01-08

**Authors:** Xin Zheng, Junxiang Hao, Hengyan Xie, Wenbao Xu

**Affiliations:** 1 College of Engineering, Heilongjiang Bayi Agricultural University, Daqing 163319, China; jwckw2021@byau.edu.cn (J.H.); xiehy555@byau.edu.cn (H.X.); 2 College of Civil Engineering and Water Conservancy, Heilongjiang Bayi Agricultural University, Daqing 163319, China; xuwenbao@byau.edu.cn

**Keywords:** bionic, pressing roller, drag reduction effect, anti-adhesion characteristic, structural optimization

## Abstract

In response to the challenges posed by high operational resistance and significant soil adhesion faced by traditional pressing rollers in moist clay soils, this study introduces a bionic pressing roller inspired by the imbricated scale structure of the pangolin. The fundamental working principles of the roller are elucidated, and its key structural parameters are designed. Utilizing the discrete element method (DEM), the structural parameters of the bionic scales are optimized through Response Surface Methodology (RSM), with traveling resistance and the mass of adhered soil serving as evaluation indicators. Field experiments are conducted to validate the operational performance of the bionic roller. The optimal parameter combination is identified as follows: a scale length of 130 mm, 10 scales, and an overlap rate of 50%. Field comparison tests reveal that the bionic roller significantly reduces traveling resistance by 11.0% and decreases the mass of adhered soil by 47.2% compared to the traditional roller at a soil moisture content of 35%. This study confirms that the bionic roller, which mimics the pangolin scale structure, demonstrates superior anti-adhesion and drag-reduction characteristics. The findings are anticipated to provide a reference for the energy-efficient design of soil-engaging components in agricultural machinery, including ridging and shaping machines.

## 1. Introduction

Soil covering and compaction are essential steps in seeding operations, as their quality directly affects seed germination, seedling growth, and crop yield [1]. The pressing roller, a key soil-engaging component in planters or ridging and shaping machines, plays a critical role in firming the soil, enhancing seed-soil contact, and improving moisture retention [2,3,4]. However, traditional steel pressing rollers encounter significant challenges in clayey or moist soil conditions, including substantial soil adhesion, high operational resistance, and excessive energy consumption, all of which negatively impact the quality and efficiency of agricultural operations [5]. Research has shown that soil adhesion can result in over a 30% increase in tillage resistance, a 30–50% rise in energy consumption, and a 5–10% decrease in seedling emergence rates [6,7]. The underlying mechanism of this adhesion phenomenon involves the formation of a continuous water film between soil particles and the metal surface due to moisture, which generates considerable capillary bridge forces and adhesion [8,9,10]. This microscopic mechanical interaction can be elucidated by the theory of liquid bridges [11]. Consequently, the development of efficient compaction components that minimize adhesion and resistance is vital for advancing precision agriculture and energy-efficient seeding technologies [12].

Bionics has recently offered a robust theoretical foundation and practical approach for enhancing the design of soil-engaging components, evolving beyond just reducing adhesion and resistance to systematically improving soil disturbance and fragmentation performance. Lu et al. [13] devised a star-toothed concave disc soil-covering and pressing device (STCP) inspired by dung beetles’ prominent head structure. Field trials revealed that this device enhanced soil-covering uniformity by approximately 18.5% in typical working conditions, effectively boosting seedbed quality. Jia et al. [14,15] created an elastic pressing roller inspired by the flexible surface morphology of earthworms. Comparative tests demonstrated that their design reduced soil adhesion mass by 32% compared to rigid rollers, significantly enhancing operational cleanliness. Zhang et al. [16] developed an auxiliary soil-breaking device modeled after the claw-toe geometry of the oriental mole cricket. In heavy clay soil, their device increased the soil breakage rate by up to 26.8%, effectively addressing the inadequate soil fragmentation capability of strip-till machines. In the realm of studying interfacial adhesion mechanisms, Qian et al. [17] optimized a metal surface based on dung beetles’ convex hull structure, effectively diminishing the liquid bridge force between wet rice leaves and the metal surface by balancing atmospheric pressure. This optimization offers a crucial theoretical framework for comprehending and managing interfacial adhesion. Furthermore, Lin et al. [18] designed a bionic subsoiler based on the geometric configuration of mole forepaws, which achieved approximately a 15.7% reduction in working resistance in field tests, demonstrating excellent drag-reduction performance. Torotwa et al. [19] similarly developed a biomimetic rotary tiller blade inspired by the claw-toe morphology of moles, as illustrated. The results demonstrated that the bionic blade achieved a 21.05% reduction in torque, effectively fragmented soil into smaller aggregates, and significantly improved straw burial rate and seedbed quality post-operation. Akter et al. [20] incorporated both longitudinal and vertical cross-sectional profiles of claw toes to design biomimetic excavator bucket teeth, integrating characteristics from canine claws and badger paw toes, which exhibited superior drag reduction performance. Naziri et al. [21] engineered an earthworm-inspired subsurface penetration probe and conducted experiments using lunar regolith simulants. Their findings revealed a maximum peak power requirement of 0.2 watt-hours at a constant penetration rate of 0.2 cm/s, accompanied by an 80% reduction in penetration resistance.

Despite significant advancements in bionic design and performance enhancement, much of the research has focused on structures such as convex hulls, corrugations, teeth, or claw toes to improve cutting or anti-adhesion properties [22,23,24]. However, there has been a notable deficiency in systematic investigations into the potential applications of the unique imbricated (overlapping) arrangement of pangolin scales for the anti-adhesion design of pressing rollers. Previous studies on anti-adhesion rollers have primarily concentrated on modifications to surface morphology or localized protrusions, which mainly address static or quasi-static adhesion mechanisms [25,26]. In contrast, pangolin scales feature a dynamic overlapping configuration that not only reduces the real contact area but also actively disrupts the continuity of the interfacial water film through micro-displacements during soil–tool interaction, thereby offering a fundamentally different mechanism for adhesion mitigation. The modeling of soil-tool interactions has increasingly utilized discrete numerical methods that accurately represent the granular nature of agricultural soils. While the Discrete Element Method (DEM) has established itself as a standard tool for simulating soil-engaging components, recent advancements in discrete mechanical modeling provide complementary insights. Notably, the kinematic swarm-based approach developed by Rapisarda and dell’Erba [27] simulates material deformation through the repositioning of discrete particles, governed by local interaction rules, without relying on explicit force or energy concepts. Their model illustrates how emergent macroscopic behaviors, such as ductile, plastic, and fracture responses, can result from simple geometric rules among neighboring points. This methodology shares fundamental conceptual similarities with DEM in its treatment of discrete interactions, indicating the potential for cross-fertilization between biomimetic design and advanced discrete modeling techniques.

To address this research gap, this paper presents a bionic pressing roller characterized by scale-like structures inspired by the imbricated morphology of pangolin scales. Unlike existing designs that primarily employ convex hulls, corrugations, or elastic surfaces to reduce adhesion, this study introduces a dynamically overlapping scale arrangement that actively disrupts the soil–roller interface. The design capitalizes on the unique ability of staggered rigid scales to induce micro-displacements in soil, which periodically breaks liquid bridges and interrupts the formation of continuous water films, marking a mechanistic shift from conventional static or morphology-dependent approaches. Additionally, this work establishes a comprehensive methodology that integrates 3D reconstruction of biological prototypes with Discrete Element Method (DEM) simulations and Response Surface Methodology (RSM) for systematic parameter optimization. The structural parameters of the bionic roller will be optimized through DEM and RSM, followed by field experiments to validate its performance. This study aims to provide innovative bionic solutions and a theoretical foundation for the energy-efficient design of soil-engaging components, including those utilized in ridging and shaping machines.

## 2. Materials and Methods

### 2.1. Analysis of Soil Adhesion Mechanism

Soil adhesion, a prevalent natural occurrence, presents substantial challenges in agricultural engineering. Therefore, resolving the issue of soil adhesion is crucial for enhancing production efficiency. In soil containing a specific moisture level, water rings or films develop on the surfaces of soil particles and solid materials in proximity. The adhesive force ***F*** between the soil and the contacting material can be defined by the subsequent equation:

(1) F=4πRγcosθ

where γ is the surface tension of water (N/m), θ is the contact angle (°), and *R* is the radius of the soil particle (m).

**Figure 1:**
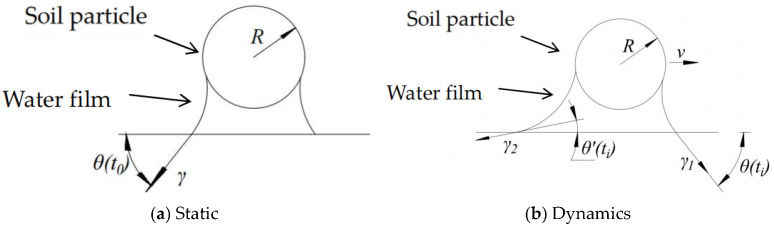
Mechanical analysis of soil particles and water film.

When a soil particle moves at a constant velocity over the surface of a soil-engaging component, sliding occurs between the water ring and the component. This motion causes a deformation of the meniscus of the water ring [28], as illustrated in the transition from the static state in Figure 1a to the dynamic state in Figure 1b.

As the velocity increases, the normal adhesive force at the interface intensifies. Consequently, the contact angle θ(*t_i_*) at the soil particle interface becomes obtuse, while the contact angle θ′(*t_i_*) at the soil-engaging component interface approaches 0°. This change in contact angles signifies a critical shift in the force balance: the horizontal component of the surface tension *γ*_2_ now acts to impede the movement of the soil particle, while simultaneously, the horizontal component of the surface tension *γ*_1_ resists the motion of the soil-engaging component.

The dynamic tangential adhesive force *F_τ_* is consequently defined as the resultant force of these two opposing surface tensions, *γ*_1_ and *γ*_2_ [29]. This can be expressed by the following equation:

(2) $$F_{\tau }=l_{2}\gamma_{2}\cos\theta^{\prime }(t_{i})-l_{1}\gamma_{1}\cos\theta (t_{i})$$

where *l*_1_ is the acting length related to the movement of surface tension $\gamma_{1}$ (m), and *l*_2_ is the acting length related to the movement of surface tension $\gamma_{2}$ (m).

To simplify the theoretical model for analyzing the dynamic interaction at the soil-tool interface, a standard assumption is applied where the acting lengths are considered equal ($l_{1}$ = $l_{2}$ = l), and the surface tension values are identical ($\gamma_{1}$ = $\gamma_{2}$ = γ). This assumption is valid for modeling a uniform interfacial water film. Substituting these conditions into Equation (2) yields a streamlined expression for the dynamic tangential adhesive force:

(3) $$F_{\tau }=\gamma l\cos\theta^{\prime }(t_{i})-\gamma l\cos\theta (t_{i})$$

**Figure 2:**
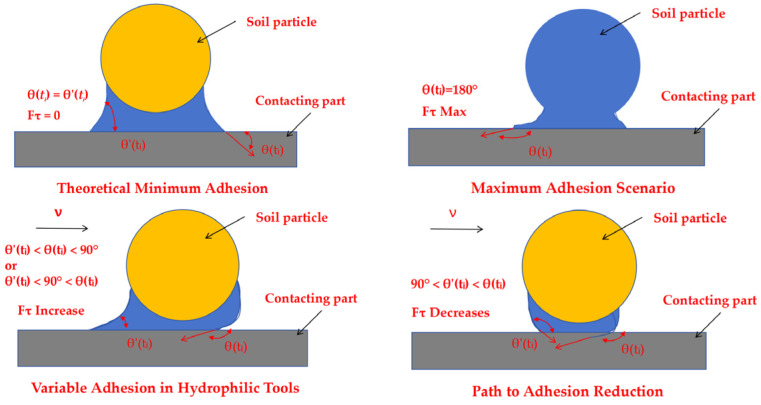
Diagram of inequality cases.

The interfacial adhesion system is inherently dynamic. Due to the hysteresis of the water film’s motion, the contact angle at the soil particle interface, θ(*t_i_*), is always greater than the angle at the tool interface, θ′(*t_i_*). This fundamental inequality leads to several critical scenarios in Figure 2:

Theoretical Minimum Adhesion: The hypothetical case where θ(*t_i_*) = θ′(*t_i_*) would, according to Equation (3) from our previous discussion, result in a tangential adhesive force *F_τ_* = 0, leaving only the normal adhesive component. However, this equilibrium state is unattainable in practice due to the inherent hysteresis.

Maximum Adhesion Scenario: When the soil particle is completely wetted (θ(*t_i_*) = 180°) and the tool surface is strongly hydrophilic (θ′(*t_i_*) = 0°), the tangential adhesive force reaches its maximum value. This represents the worst-case scenario for soil adhesion.

Variable Adhesion in Hydrophilic Tools: For tools made of hydrophilic materials like steel, the condition θ′(*t_i_*) < θ(*t_i_*) < 90°or θ′(*t_i_*) < 90° < θ(*t_i_*) is common. Here, both angles change dynamically as the interface moves, causing *F_τ_* to fluctuate significantly over time. This explains the unstable and high adhesion observed with conventional materials.

Path to Adhesion Reduction: The most desirable condition for reducing adhesion is when 90° < θ′(*t_i_*) < θ(*t_i_*). Crucially, the tangential force *F_τ_* decreases as the rate of change of θ(*t_i_*). A larger differential in these rates leads to a greater reduction in adhesive force. This principle underpins the effectiveness of anti-adhesion designs. The introduction of bionic scale structures, for instance, disrupts the formation of continuous water films and promotes a rapid change in the effective contact angle at the soil interface, thereby significantly lowering adhesion [30,31].

### 2.2. Surface Structure Extraction Method of Pangolin Scales

**Figure 3:**
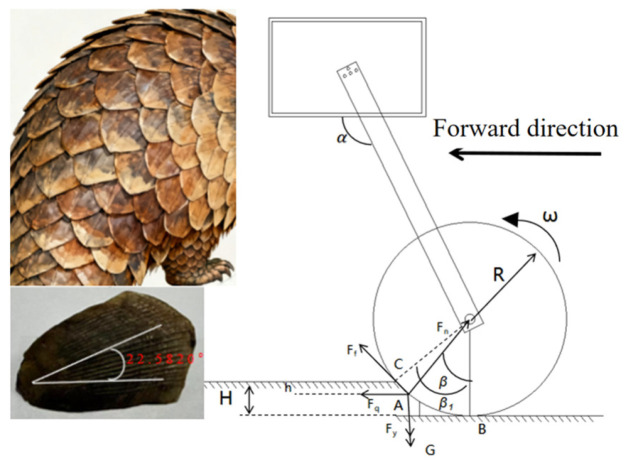
Comparison of the contact angle between the structure of pangolin scales and the pressing roller.

As shown in Figure 3, the optimal incidence angle of the pangolin scales contacting the soil is about 22.5, which is close to the angle of the pressing roller and the soil. Based on the analysis above, this paper reconstructs the structure of the pangolin scales by using the reverse engineering technology, and designs the bionic scales with the characteristics of water film blocking as shown in Figure 3 [32].

**Figure 4:**
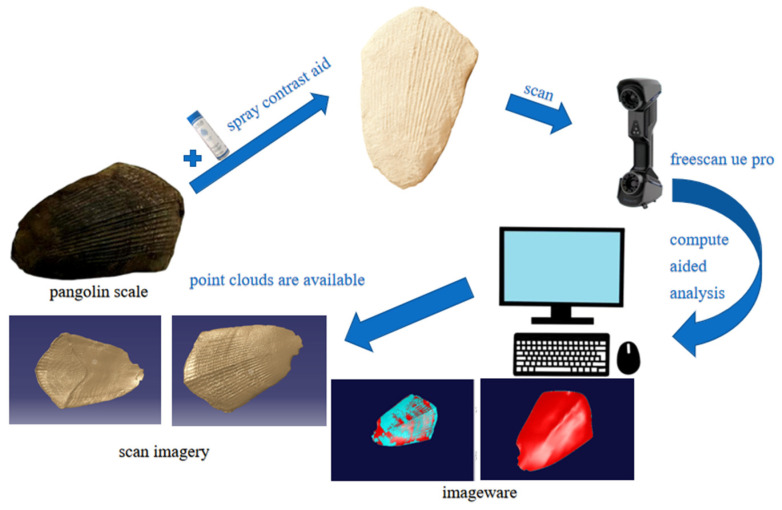
Process of point cloud acquisition and checking.

Initially, the sample surface was cleaned with a 50% alcohol solution to eliminate contaminants. The surface morphology was subsequently examined using an ultra-deep field scanning electron microscope (Opton Optical Technology Co., Ltd., Beijing, China), and key parameters of the curved edges were measured [33]. To reduce reflections and data distortion from ambient light, an FC-5 contrast enhancer was uniformly applied to the sample surface before scanning [34]. Point cloud data of the scale surface was then collected using a Freescan UE Pro 3D scanner (Xianlin 3D Technology Co., Ltd., Hangzhou, China). The acquired data underwent comprehensive preprocessing, which included multi-view registration and noise filtering, to ensure high fidelity. Based on this processed data, a precise three-dimensional model of the scale was reconstructed using reverse engineering methodologies. The entire reconstruction process was rigorously controlled to maintain an error margin within ±5% [35], thereby ensuring the model’s accuracy and reliability. The experimental workflow is depicted in Figure 4.

### 2.3. Design and Analysis of the Pressing Roller Based on Pangolin Scale Structure

**Figure 5:**
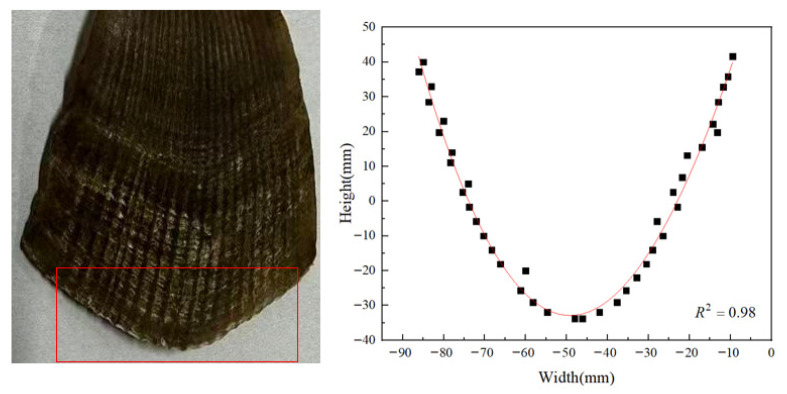
Scale contour fitting curve.

To determine the structural parameters of the pangolin scale, parametric curves were extracted. The 3D model was imported into Solid Works 2025, where sequential point selection and coordinate data measurement were conducted. Subsequently, the data were fitted using Origin 2018 software to derive the contour fitting curve and the corresponding fitting equation for an individual pangolin scale surface. The resulting fitting curve is illustrated in Figure 5.

Pangolin scales display an imbricated arrangement characterized by polygonal contours, curved edges, and a flexible yet rigid connection mechanism. This distinctive structure allows for micro-displacements during movement through soil, effectively dislodging adhered soil particles. In this study, the dimensions of a conventional pressing roller, with a total length of 80 cm and a diameter of 90 cm, served as the baseline reference. The scale structure was simplified, arranged in a staggered horizontal connection. Key structural parameters were defined, including scale lengths of 110 mm, 130 mm, and 150 mm; numbers of scales of 8, 10, and 12; and overlap ratios of 40%, 50%, and 60%. By integrating fitted equations derived from the geometric characteristics of the pangolin scale and its contour curves, a three-dimensional model of the bionic roller was constructed, as shown in Table 1.

### 2.4. Numerical Simulation Method of Bionic Roller Based on EDEM

Soil exhibits discrete structural characteristics. A coupled simulation model of the component-soil system was developed based on the Discrete Element Method (DEM) using EDEM 2024 software [36,37]. This model simulated the field operation of the bionic pressing roller under conventional working conditions, with the traveling resistance of the roller and the mass of adhered soil as the key evaluation indicators. The primary factors affecting the device’s performance were analyzed through this simulation.

The study accounted for the elastic-plastic contact deformation resulting from the extrusion and collision between soil particles. The EEPA model was selected to analyze the cohesive force due to soil moisture and the linear viscoelastic deformation during the roller’s operation [38]. Furthermore, given its effectiveness in reducing adhesion and resistance during the compaction of cohesive soils, the Hertz-Mindlin model was adopted as the contact force model for both soil-soil and soil-pressing roller interactions [39,40].

**Figure 6:**
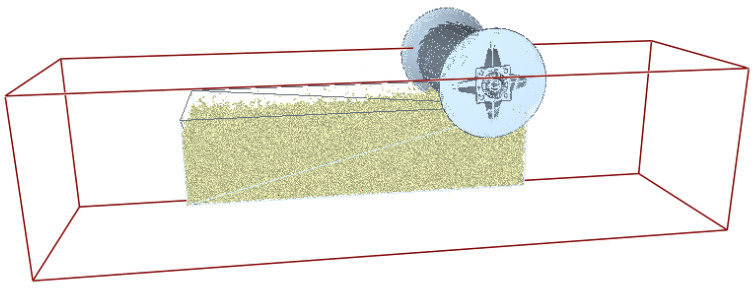
Simulation model of soil trough and bionic pressure roller.

To accurately reflect the actual conditions in the numerical simulation, soil particles were sieved using a four-level Taylor sieve, resulting in the particle size distribution and mass percentages shown in Table 2. The 3D model of the bionic pressing roller prototype was imported into EDEM2024 software in .stl format, and a simulated soil bin with dimensions of 1500 mm × 500 mm × 500 mm was established, as illustrated in Figure 6.

As shown in Table 3, the soil and other parameters of the compaction roller are displayed. Based on this, the actual model of the target soil trough and compaction roller is established.

This study utilized the Box–Behnken experimental design method to systematically examine the influence of key structural parameters on the performance of the bionic pressing roller. The evaluation indicators selected were traveling resistance (Y_1_) and mass of adhered soil (Y_2_). A series of experiments was conducted by varying three critical factors: scale length (coded value X_1_), number of scales (coded value X_2_), and scale overlap ratio (coded value X_3_). The levels and corresponding coded values for these experimental factors are detailed in Table 4. A summary of the experimental design and the corresponding results is provided in Table 5.

### 2.5. Simulation Parameter Calibration and Boundary Conditions

To ensure the accuracy of the simulations, soil model parameters were calibrated using laboratory tests that replicated field conditions. Soil samples collected from the test field at a depth of 0–20 cm were characterized for moisture-density relationships via standard Proctor compaction tests (ASTM D698). The specific soil parameters employed in the discrete element method (DEM) simulations were validated through direct shear tests (ASTM D3080) conducted at moisture contents of 15%, 25%, and 35%. These tests provided calibrated values for cohesion and the internal friction angle that accurately reflect actual field conditions [41].

Spatial variability within the test field was examined through systematic sampling conducted across a 10 × 10 m grid prior to the experiments. Measurements of soil compactness, obtained using a SC-900 soil compactness tester, revealed variations within ±15% of the mean value. The experimental pathway was chosen to reflect the average conditions of the field. This spatial characterization ensured that the simulation boundary conditions accurately represented the heterogeneity of the field [42].

The discrete element method (DEM) simulation incorporated realistic boundary conditions by employing a multi-layer soil generation approach. Soil particles were generated according to a normal distribution of sizes, as detailed in Table 2, with randomized initial positions within the soil bin. The bottom boundary utilized a fixed wall condition to simulate the natural soil foundation, while the side boundaries implemented periodic boundary conditions to reduce edge effects. The roller-soil interaction parameters, presented in Table 3, were validated by comparing them with experimental force measurements from preliminary tests, thereby ensuring that the contact mechanics accurately represented the behavior of the soil-tool interface.

Soil preparation procedures in the simulations closely mirrored field practices. The initial generation of particles simulated loose soil conditions resulting from tillage, followed by a settling period under gravity to establish a realistic initial density. This multi-step approach ensured that the initial conditions of the simulation accurately represented the actual state of the field prior to roller engagement.

### 2.6. Field Test Method of Bionic Pressing Roller

**Figure 7:**
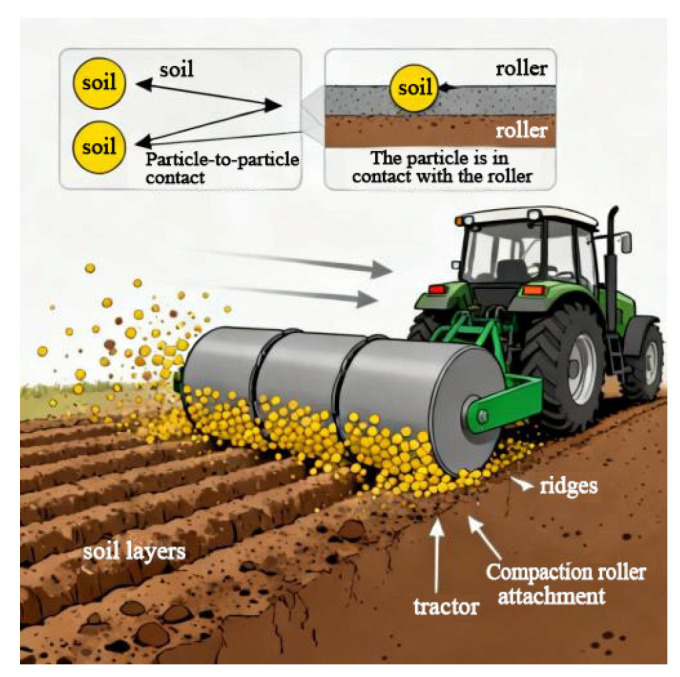
Schematic diagram of the adhesion mechanism of the pressing roller.

As shown in Figure 7, during operation, the interplay between soil particles and the roller surface creates a continuous water film, resulting in soil adhesion. This phenomenon increases the roller’s travel resistance, thereby reducing operational efficiency.

**Figure 8:**
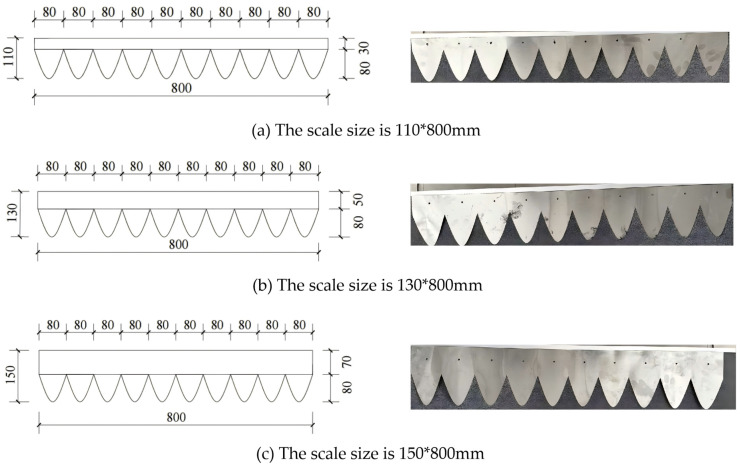
Bionic scale sample.

The bionic scale material is made of 304 stainless steel, as shown in Figure 8. The samples are processed by Guangzhou Manlong Sheet Metal Factory (Guangzhou, China) and connected by bolts. Based on the discrete element simulation results, the relatively superior bionic structure designs F-1 (130 mm, 50%, 10), F-2 (110 mm, 60%, 10), and F-3 (150 mm, 60%, 10) were selected to process three different bionic scales, as shown in Figure 7. Taking the traveling resistance and the quality of adhered soil as the test indicators, a comparative analysis was conducted with the traditional pressing roller (DZ) to further explore the rationality of the structural design of the bionic pressing roller and its impact on reducing soil adhesion.

Field experiments were conducted in October 2025 at Jianshan Farm in Nenjiang City, Heilongjiang Province. The experimental setup employed a John Deere 7M-2204 tractor (John Deere (Tianjin) Co., Ltd., Tianjin, China) equipped with a ridging machine. Monitoring instruments included a high-precision counting scale (0.1 g accuracy), a spoke-type tension-compression sensor (0–3 t range), a vernier caliper (0.02 mm accuracy), a soil core sampler, a four-level Taylor sieve set, a soil compactness tester, and a thermostatic drying oven. Pre-experiment measurements were taken five times, with averaged values recorded as baseline data. The test field featured soybean as the preceding crop, with a roller length of 1.1 m operating at a speed of 5 km/h and under a soil moisture content of 24.7%.

**Figure 9:**
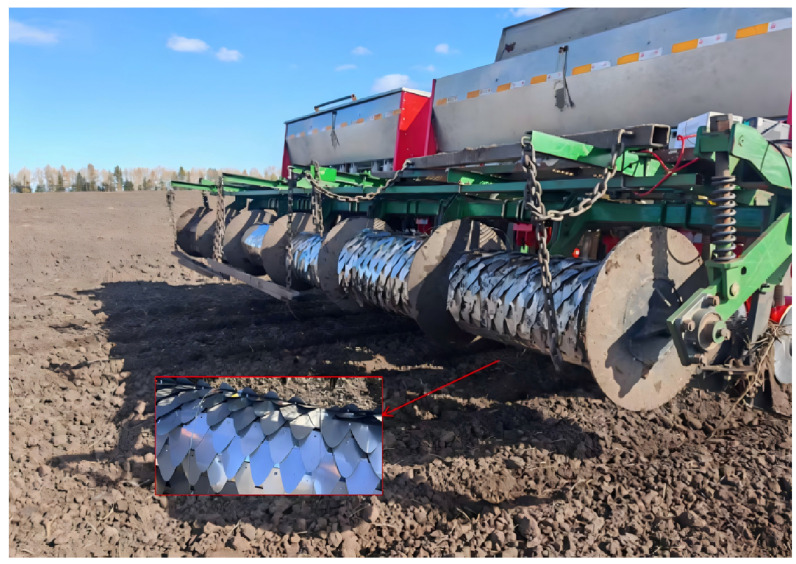
Field test.

**Figure 10:**
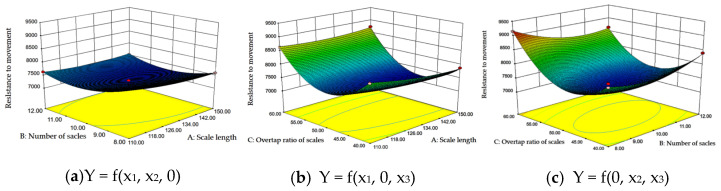
Response surfaces of interactive factors influence on test indexes.

**Figure 11:**
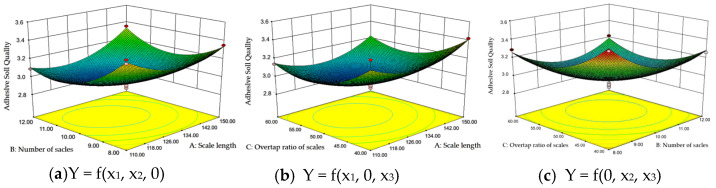
The interaction factors respond to the influence of adhering soil quality.

As shown in Figure 9, the scale material used in the field experiment was fabricated from 304 stainless steel. The samples are processed by Guangzhou Manlong Sheet Metal Factory and connected by bolts. Based on the discrete element simulation results, the relatively superior bionic structure designs F-1 (130 mm, 50%, 10), F-2 (110 mm, 60%, 10), and F-3 (150 mm, 60%, 10) were selected to process three different bionic scales, as shown in Figure 10 and Figure 11. Taking the traveling resistance and the quality of adhered soil as the test indicators, a comparative analysis was conducted with the traditional pressing roller (DZ) to further explore the rationality of the structural design of the bionic pressing roller and its impact on reducing soil adhesion.

The average mass of soil adhesion for each test group was determined from five repeated measurements using a high-precision analytical balance. The traveling resistance was measured by a spoke-type tension-compression load cell. The corresponding experimental data are summarized in Table 6.

## 3. Result and Discussion

### 3.1. Adhered Soil Mass and Traveling Resistance Analysis Based on Response Surface Method Analysis

According to the results presented in Table 7, the regression model for traveling resistance (Y_1_) demonstrates a highly significant level, with a *p*-value of less than 0.0001. The lack-of-fit term, which has a *p*-value of 0.2382 (greater than 0.05), is not significant, indicating minimal interference from uncontrollable factors and confirming the appropriateness of the selected model. All three primary factors exhibit significant effects on the experimental results. Among these, Factor ***C*** (scale overlap rate) exerts the most substantial influence, as evidenced by its exceptionally high F-value of 406.76, which far exceeds those of Factors ***A*** and ***B***. Additionally, the interaction effect between Factors ***B*** and ***C*** (the number of scales and the overlap rate) is both substantial and highly significant. This finding underscores that the combined effect of the number of scales (***B***) and the overlap rate (***C***) on the results is greater than the sum of their individual effects. The regression equation relating the factors to the response indicator is derived as follows.

(4) $$\begin{array}{l} Y=7281.9-89.28A-72.62B+320.48C+53.02AB+112.46AC\\ -200.77BC+90.82A^{2}+300.98B^{2}+995.21C^{2} \end{array}$$

A fitting analysis of the experimental results yielded the analysis of variance (ANOVA) table for the adhered soil mass, as shown in Table 7. The results indicate that the model is highly significant (*p* < 0.01), confirming the appropriateness of the selected model and demonstrating a definitive relationship between the experimental factors and the response variable. The order of influence of the factors and their interactions, from greatest to least, is as follows: ***B*** > ***A***^**2**^ = ***C***^**2**^ > ***B***^**2**^ > ***C*** > ***AB*** > ***BC*** > ***AC*** > ***A***. All factors are significant, with scale length (***B***) and overlap rate (***C***) exhibiting the most pronounced effects on adhesion mass among the main factors. The interaction between the number of scales and scale length (***AB***) has the greatest impact on adhered soil mass. The regression equation relating the factors to the response variable is as follows:

(5) $$\begin{array}{l} Y=2.95+0.035A-0.081B-0.094C+0.08AB-0.005AC\\ -0.013AB+0.048BC+0.15A^{2}+0.19B^{2}+0.18C^{2} \end{array}$$

Response surface analysis was performed to examine the interaction effects of various factors on the evaluation indicators. In the regression equation, one factor was maintained at the zero level (center point) while the influence of the other two factors on traveling resistance was assessed. Utilizing Design-Expert 10.0 software, the interaction effects on the response surface were illustrated in Figure 10.

Analysis of Figure 10b indicates that, with a constant number of scales, traveling resistance initially decreases and subsequently increases as the overlap rate rises. As the number of scales increases, traveling resistance becomes more favorable within the scale length range of 126 mm to 134 mm and an overlap rate of 45% to 50%.

In Figure 10c, when the scale length is held constant, traveling resistance exhibits a similar trend of initial decrease followed by an increase with rising overlap rate. The most favorable traveling resistance occurs when the number of scales is between 9 and 10 and the overlap rate is between 45% and 50%. Under these conditions, the contour lines of the response surface form closed ellipses, indicating a significant interaction between the factors influencing traveling resistance and the presence of a minimum value within the design space.

Response surface analysis was utilized to examine the interaction effects of various factors on the adhered soil mass. By fixing one factor at the zero level and analyzing the remaining two factors, the interaction effects were systematically assessed using Design-Expert 10.0 software, as depicted in Figure 11.

Analysis of Figure 11a reveals that when the scale overlap rate is held constant, the adhered soil mass initially decreases before subsequently increasing with variations in the other factor. Optimal performance in reducing adhered soil mass occurs when the scale length is between 126 mm and 134 mm, combined with 9–10 scales.

In Figure 11b, when the number of scales is held constant, the adhered soil mass exhibits a similar trend of initial decrease followed by an increase as the overlap rate changes. The most favorable conditions arise when the scale length is within 126–134 mm and the overlap rate ranges from 45% to 50%.

Figure 11c demonstrates that when the scale length is fixed, the adhered soil mass again displays a characteristic pattern of decreasing and then increasing with an increasing overlap rate. The best results are obtained with 10–11 scales and an overlap rate of 45–50%. The closed elliptical contour lines in this response surface indicate a significant interaction between the factors influencing adhered soil mass and confirm the presence of a minimum value within the design space.

Based on the operational performance requirements of the bionic pressing roller and the practical working conditions, ridging and compaction operations must achieve low traveling resistance and minimal soil adhesion. To optimize these competing objectives, a multi-criteria optimization was conducted, utilizing traveling resistance and adhered soil mass as the target functions. The design variables included three experimental factors: scale length, number of scales, and scale overlap rate. The optimization constraints were established as follows:

Through a comprehensive analysis of the influence patterns of three experimental factors on traveling resistance and adhered soil mass, the optimal parameter combination was identified via numerical optimization using Design-Expert 10.0 software. This optimization process yielded the following optimal working parameters: a scale length of 126.52 mm, a number of scales of 9.44, and a scale overlap rate of 48.05%.

To enhance practical application in field operations, the optimized parameters were rounded to the nearest practical values: a scale length of 130 mm, a number of scales of 10, and a scale overlap rate of 50%. Three replicate validation experiments were conducted with these practical parameters, resulting in an average traveling resistance of 7438.67 N and an adhered soil mass of 2.83 kg.

The close alignment between the experimental results and model predictions demonstrates the accuracy of the regression model and validates the effectiveness of response surface methodology for parameter optimization. This validation confirms that the optimized parameters retain their performance when applied under actual working conditions.

The optimization process utilized a desirability function approach to balance the dual objectives of minimizing both traveling resistance and soil adhesion. The high degree of agreement between predicted and experimental values, with deviations of less than 5%, further substantiates the robustness of the optimization methodology.

### 3.2. Analysis of Field Test Results

**Figure 12:**
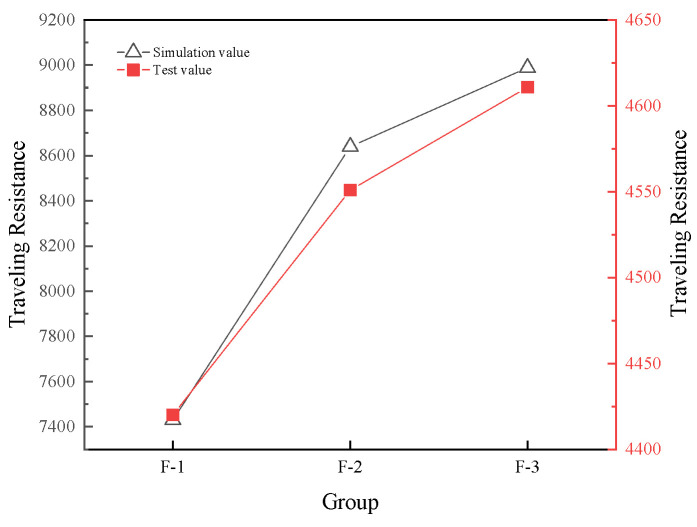
Simulation and test results of traveling resistance.

**Figure 13:**
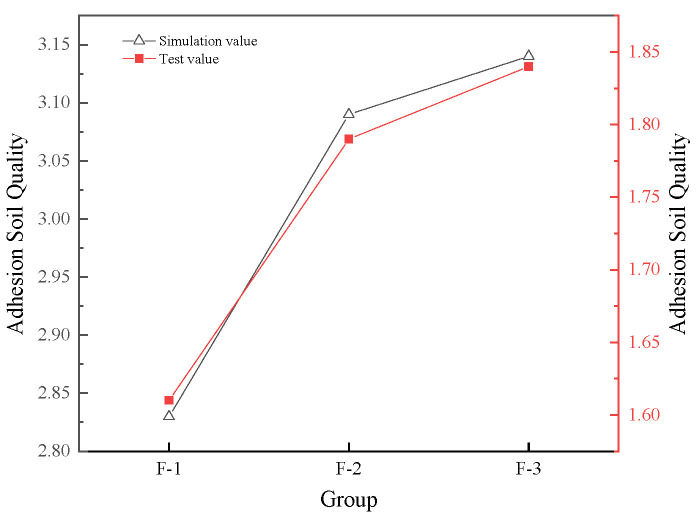
Simulation and test results of adhered soil quality.

As shown in Figure 12 and Figure 13, when the 25% moisture content condition set in the simulation is compared with the field test data under the same moisture content, the results show that the two are consistent in the changing trend, jointly verifying the regular influence of the bionic scale structure parameters on the viscosity reduction and resistance reduction effect. However, the absolute values of the traveling resistance and adhered soil mass obtained from the simulation are both higher than those measured in the field. This is mainly due to the following reasons: The ideal boundary conditions in the EDEM simulation fail to fully reflect the energy dissipation in actual field operations, including the complex interactions between soil particles and crop residues. Meanwhile, the simulation model simplifies the actual drag reduction mechanism of the dynamic disruption of water film continuity by scales and the truncation of liquid bridge formation, resulting in an overestimation of the interfacial adhesion force. It is worth noting that under the parameter combination of a scale length of 130 mm, a quantity of 10, and an overlap rate of 50%, both the simulation and experiments demonstrated the optimal performance in reducing viscosity and resistance, verifying the effectiveness of the parameter optimization. Future research can further enhance the accuracy of simulation prediction by refining the soil constitutive model and introducing more accurate dynamic contact algorithms.

The reasonable concordance between simulation and experimental trends (Figure 12 and Figure 13) validates the calibrated discrete element method (DEM) parameters and boundary conditions outlined in Section 2.5. The systematic overestimation of absolute values in simulations, which are approximately 15–20% higher than field measurements, can be ascribed to the idealization of boundary conditions and the simplified representation of soil-straw interactions in the current model. Future implementations may benefit from integrating kinematic swarm concepts [27] to more accurately capture the complex soil flow patterns surrounding the scale structures.

**Figure 14:**
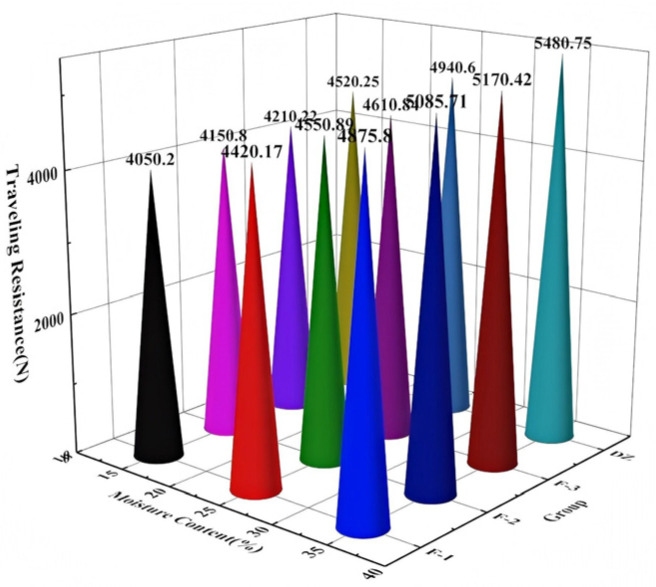
The influence of different moisture contents and groups on the traveling resistance of the pressing roller.

As illustrated in Figure 14, the traveling resistance of the three bionic treatment groups (F-1, F-2, and F-3) was significantly lower than that of the control group (DZ) across various moisture contents. Under all tested conditions, the F-1 group demonstrated the lowest resistance, exhibiting reductions of 10.4%, 10.6%, and 11.0% compared to the DZ group, thereby highlighting its consistent and superior drag-reduction performance. Moreover, although an increase in soil moisture content resulted in a rise in traveling resistance for all groups, the increments observed in the bionic groups (F-1, F-2, and F-3) were notably smaller than those in the control group. This finding suggests that the bionic designs effectively alleviated the sharp increase in resistance associated with elevated soil moisture, with the most significant advantage evident at lower moisture levels.

**Figure 15:**
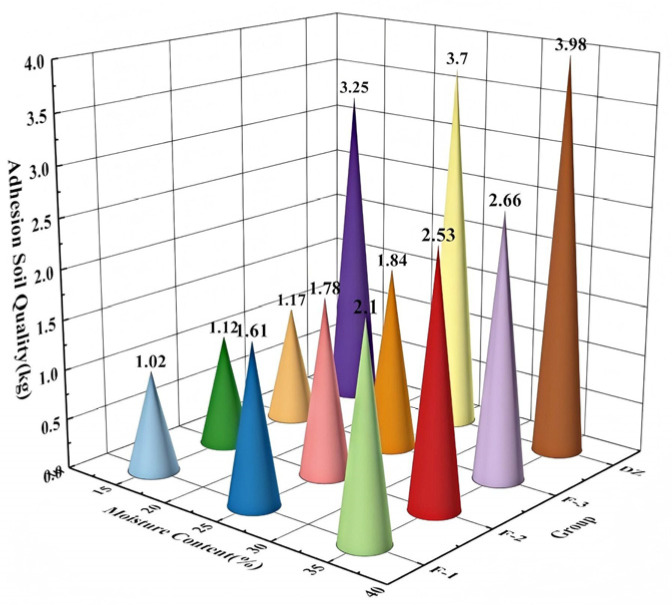
The influence of different moisture contents and groups on the amount of soil adhering to the roller.

Figure 15 illustrates that the bionic groups exhibited significantly lower soil adhesion compared to the DZ group across all moisture conditions. The F-1 group demonstrated the most effective anti-adhesion performance, achieving reductions in adhered soil mass of 68.6%, 56.5%, and 47.2% relative to the DZ group as moisture content increased from 15% to 35%. Although higher moisture content led to increased soil adhesion in all groups, the bionic groups—especially F-1—exhibited a markedly gentler slope of increase. These findings indicate that the bionic design not only substantially lowers the baseline level of soil adhesion but also reduces the sensitivity of adhesion to increasing moisture content, thereby ensuring effective and reliable anti-adhesion performance across a broad spectrum of soil moisture conditions.

## 4. Discussion

This study presents the development of a bionic pressing roller inspired by the imbricate scale arrangement of pangolin armor, which exhibited significant anti-adhesion and drag-reduction properties during field tests. Similar to the methodology employed in Reference [13], which emphasized the bionic optimization of soil-engaging components through response surface methodology, both studies are grounded in biomimetic design principles. Reference [13] introduced a convex-bump roller modeled after the head structure of dung beetles, utilizing UHMWPE material to minimize adhesion, and reported a 43.68% reduction in soil adhesion compared to a conventional VCP roller. In contrast, the current study drew from the overlapping mechanism of pangolin scales. By utilizing discrete element simulation and response surface optimization, the optimal combination of scale length, number, and overlap ratio was identified, leading to significant improvements in both traveling resistance (an 11.0% reduction) and soil adhesion (a 47.2% decrease). This reduction in resistance not only decreases fuel consumption and enhances the economic efficiency of field operations but also lessens the mechanical load on tractor transmission systems and the roller itself, thereby reducing maintenance needs and prolonging equipment service life. Additionally, the considerable decrease in soil adhesion minimizes the downtime required for cleaning during continuous operation, thereby enhancing effective working time and operational continuity, particularly in moist clay conditions, while also contributing to more uniform seedbed quality. These findings further expand the application of bionic structures in compaction components.

This study complements Lu et al.’s design concept in bionic strategy, which focused on static anti-adhesion mechanisms of surface convex structures. In contrast, our design emphasizes a “dynamic discontinuous contact” mechanism achieved through a staggered arrangement of scales that disrupts the water film continuity at the soil-roller interface during operation. Unlike Jia et al.’s elastic roller with a flexible contour-adaptation concept, our structure utilizes dynamically coupled rigid units to introduce interfacial disturbances while maintaining structural strength, ensuring effective anti-adhesion and long-term durability. From a theoretical mechanics perspective, the soil-roller interface behavior shares fundamental characteristics with cohesive interface models in fracture mechanics. Cuomo et al. [43] presented a thermodynamically consistent cohesive interface model with degrading friction coefficient, where the activation function couples normal and tangential tractions with damage energy release rate:

(6) $$g(\sigma ,\tau ,\zeta )=\frac{\tau^{2}}{\sigma_{k}^{2}}-4\mu^{2}\left(\frac{\zeta }{\zeta_{0}}-1\right)\left(\frac{\sigma }{\sigma_{k}}+\frac{\zeta }{2\zeta_{0}}-1\right)$$

where *σ* is the normal traction (Pa), *τ* is the Tangential traction (Pa), *ζ* is the energy release rate (J/m^2^), *σ*_k_ is the Characteristic stress (Pa), *ζ*_0_ is the Activation energy (J/m^2^), and *μ* is the Friction parameter (dimensionless).

This formulation captures the progressive degradation of interfacial strength with damage accumulation, a phenomenon analogous to the disruption of soil-roller adhesion in our bionic design. The model’s associated flow rule naturally incorporates dilatancy effects that diminish with damage evolution, mirroring the reduction in soil adhesion as scale-induced micro-displacements break continuous water films. While our current study focuses on macroscopic performance validation, future work could integrate such thermodynamic frameworks to develop more predictive models of interface degradation. This would enable quantitative characterization of how scale geometry parameters influence the cohesive-frictional behavior at soil-tool interfaces, potentially leading to more fundamental design criteria for anti-adhesion components. Furthermore, our scale-based configuration prioritizes macroscopic mechanical action and dynamic energy conversion over surface morphological mimicry, distinguishing it from the rib-type roller by Zhang et al. based on dung beetles’ ventral geometry.

The proposed structure, from an energy-conversion standpoint, exhibits similarities with the soil-cutting and lifting mechanism of star-toothed concave discs, both modifying the soil-tool interaction to minimize energy usage. Our design innovation lies in converting the continuous shearing process into a periodic local lifting and turning action, decreasing resistance and weakening soil adhesion. This mechanism demonstrates enhanced durability under high-speed, high-pressure conditions, rendering it more practical for real-world use than strategies relying on superhydrophobic coatings or intricate microstructures to prevent adhesion.

In summary, this study introduces a novel anti-adhesion pathway, termed the “dynamic rigid-unit array,” which builds upon established bionic design techniques like 3D reconstruction, discrete element simulation, and parameter optimization. This innovative approach enhances existing designs, such as the “star-toothed disc + convex-bump roller,” to propel the evolution of soil-engaging components towards increased efficiency, energy conservation, and longevity.

## 5. Conclusions

This study developed and analyzed a bionic pressing roller inspired by the structural characteristics of pangolin scales. The anti-adhesion and drag-reduction performance of the roller were assessed through discrete element simulations and field experiments. The findings indicate that the bionic scale structure effectively diminishes both traveling resistance and soil adhesion. The optimal parameter set derived from the experiments serves as a foundation for the enhanced design of ridge-forming machines. The bionic roller significantly decreased adhesion and resistance by disrupting the continuous water film at the soil–roller interface and minimizing the contact area through its scale structure. However, when the scale length, number, or overlap ratio exceeded the optimal range, the reduction in traveling resistance was less pronounced, and structural variations occasionally resulted in increased resistance.

(1) The bionic roller markedly decreased adhesion and resistance by disrupting the continuous water film at the soil–roller interface and minimizing the contact area through its scaled structure. However, when the scale length, number, or overlap ratio surpassed the optimal range, the reduction in traveling resistance became less pronounced; structural variations even resulted in increased resistance.
(2) Discrete element simulation and response surface methodology demonstrated the significant impact of bionic scale parameters on traveling resistance and soil adhesion mass. The optimized parameter set, rounded to practical values, comprised a scale length of 130 mm, 10 scales, and a 50% overlap ratio. Validation tests conducted under these conditions yielded a traveling resistance of 7438.67 N and an adhered soil mass of 2.83 kg, which correspond closely with model predictions.
(3) In field tests, the bionic roller demonstrated superior anti-adhesion and drag-reduction capabilities compared to the conventional roller across various soil moisture levels. The F-1 design achieved the highest performance, decreasing traveling resistance by 10.4–11.0% and reducing adhered soil mass by 47.2–68.6% relative to the DZ (conventional) roller. Furthermore, as moisture content increased, the bionic roller exhibited significantly smaller increases in resistance and adhesion than the conventional roller, suggesting that its design effectively mitigates the adhesion-promoting effects of soil moisture and accommodates a broader range of working conditions.

## Figures and Tables

**Table 1:** Three-dimensional diagrams of different bionic pressure roller scales.

		Numbers of Scales		
		8	10	12
Scale lengths (mm)	110	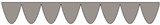	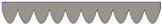	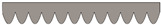
	130	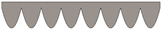	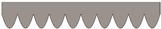	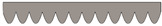
	150	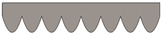	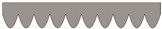	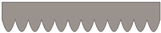

**Table 2:** Soil particle model.

Classification	Geometry	Particle Size (mm)	Proportion (%)
Chad	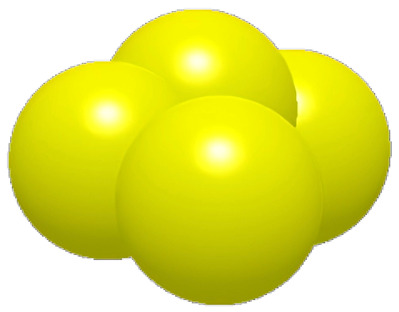	2~2.5	16.19
Sand	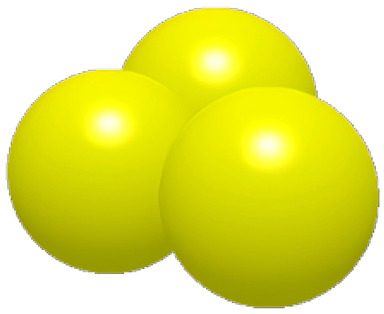	1.5~2	9.71
Silt	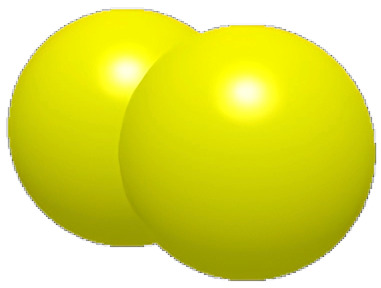	1~1.5	15.34
Cosmid	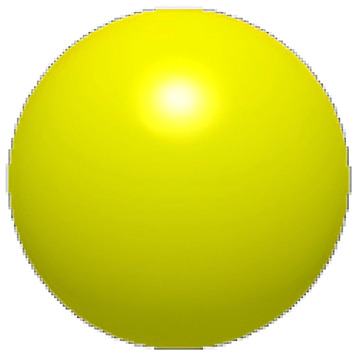	0.25~1	58.76

**Table 3:** Material properties and contact parameters.

Parameter	Unit	Numerical Value
Poisson’s ratio of soil particles	-	0.35
Shear modulus of soil particles	MPa	1.06
Density of soil particles	kg/m^3^	1540
Poisson’s ratio of pressure roller	-	0.3
Shear modulus of pressure roller	MPa	1.92
Recovery coefficient between particles	-	0.13
Rolling friction factor between particles	-	0.27
Static friction factor between particles	-	0.56
Recovery coefficient between particles and pressure roller	-	0.16
Rolling friction factor between particles and pressure roller	-	0.5
Static friction factor between particles and pressure roller	N	0.43
Acceleration of gravity	m/s^2^	9.81

**Table 4:** Experiment factor level and coded value.

Coded Value	Scale Length (mm)	Number of Scales	Scale Overlap Rate (%)
−1	110	8	40
0	130	10	50
1	150	12	60

**Table 5:** Simulation orthogonal experiment scheme and results.

No.	Scale Length X_1_ (mm)	Number of Scales X_2_	Scale Overlap Rate X_3_ (%)	Y1 (N)	Y2 (kg)
1	0	0	0	7219.47	2.89
2	−1	1	0	7861.28	3.19
3	0	1	−1	8404.72	3.26
4	0	−1	1	9153.00	3.29
5	−1	−1	0	7937.13	3.41
6	0	0	0	7304.51	2.9
7	1	−1	0	7391.85	3.35
8	0	0	0	7279.62	2.87
9	0	−1	−1	8297.92	3.52
10	1	0	1	8988.51	3.09
11	0	0	0	7294.88	2.92
12	1	1	0	7516.32	3.35
13	−1	0	−1	7972.61	3.39
14	−1	0	1	8639.66	3.14
15	0	1	1	8456.02	3.22
16	0	0	0	7311.03	3.19
17	1	0	−1	7871.27	3.42

**Table 6:** Experiment scheme and results.

No.	Moisture Contents (%)	Test Number	Traveling Resistance (N)	Adhering Soil Quality (kg)
1	15	F-1	4050.2	1.02
2	25	F-1	4420.17	1.61
3	35	F-1	4875.8	2.1
4	15	F-2	4150.8	1.12
5	25	F-2	4550.89	1.78
6	35	F-2	5085.71	2.53
7	15	F-3	4210.22	1.17
8	25	F-3	4610.84	1.84
9	35	F-3	5170.42	2.66
10	15	DZ	4520.25	3.25
11	25	DZ	4940.6	3.7
12	35	DZ	5480.75	3.98

**Table 7:** Analysis of variance for traveling resistance Y_1_ and adhered soil quality Y_2_.

	Sum of		Mean	F	*p*-Value
Source	Squares (Y1/Y2)	df	Square (Y1/Y2)	Value (Y1/Y2)	Prob > F (Y1/Y2)
Model	5.96× *× 10^−6^/0.78	9	6.6× * 10^−5^/2.2× * 10^−3^	327.99/10.18	<0.0001 ***/<0.0001 ***
A-Scale length	63,763.78/0.002	1	63,763.78/0.2	31.57/925.01	0.0008 ***/0.0153 **
B-Number of scales	44,540.68/0.2	1	44,540.68/0.037	22.05/169.16	0.0022 ***/<0.0001 ***
C-Scale overlap rate	8.2× * 10^−5^/0.037	1	8.2× * 10^−5^/0.026	406.76/118.05	<0.0001 ***/<0.0001 ***
AB	11,245.54/0.026	1	11,245.54/5.9× * 10^−3^	5.57/27.26	0.0504 */<0.0001 ***
AC	50,591.26/0.006	1	50,591.26/9.0× * 10^−3^	25.05/41.62	0.0016 ***/0.0012 ***
BC	1.6× * 10^−5^/0.009	1	1.6× * 10^−5^/0.18	79.82/821.97	<0.0001 ***/0.0003 ***
A^2^	34,728.80/0.18	1	34,728.8/0.15	17.19/685.29	0.0043 ***/<0.0001 ***
B^2^	3.8× * 10^−5^/0.15	1	3.8× * 10^−5^/0.18	188.83/818.18	<0.0001 ***/<0.0001 ***
C^2^	4.1× * 10^−6^/0.18	1	4.1× * 10^−6^/4.7× * 10^−4^	2064.52/23.97	<0.0001 ***/<0.0001 ***
Residual	14,139.73/0.001	7	2019.96/2.2× * 10^−3^	-	-
Lack of Fit	8708.78/× * 10^−5^	3	2902.93/× * 10^−5^	2.14/10.18	0.2382/0.0051
Pure Error	5430.95/0.78	4	1357.74/-	-	-
Cor Total	5.97× * 10^−6^/0.78	16	-	-	-

*** indicates extremely significant (*p* < 0.01); ** indicates significant (0.01 < *p* < 0.05); * indicates significant (0.05 < *p* < 0.1).

## Data Availability

The original contributions presented in this study are included in the article. Further inquiries can be directed to the corresponding author.

## References

[1] Song X., Zhang F., Dai F., Chen H., Zhao W. (2025). Research progress on the simulation model of cultivated loessial soil and anti-sticking and resistance reduction technology of soil-engaging components. Trans. CSAE.

[2] Liu D., Guo Z., Wang X., Lu J., Qiao H. (2023). Design and Test of Ultra Short and Low Orchard Management Machine. Res. Agric. Mech..

[3] Xu T., Zhang R., Wang Y., Jiang X., Feng W., Wang J. (2022). Simulation and Analysis of the Working Process of Soil Covering and Compacting of Precision Seeding Units Based on the Coupling Model of DEM with MBD. Processes.

[4] Acquah K., Chen Y. (2021). Discrete Element Modelling of Soil Compaction of a Press-Wheel. AgriEngineering.

[5] Qian D., Zhang J. (1984). Research on adhesion and friction of soil against metallic materials. Acta Agromech.

[6] Liu G., Xia J., Zheng K., Cheng J., Wei Y. (2022). Design and experiments of the barrier type rotary anti-adhesion blade roller with vibration crosspiece. Trans. CSAE.

[7] Zheng K. (2019). Research status and prospect of soil anti-adhesion technology for tillage equipment. J. Anhui Agric. Univ..

[8] Li Y.F., Hu J., Li Q., Kong Q.L. (2018). Research on the design and imptovement of IZQ-440 Type Ridger. J. Shanxi Agric. Univ. (Nat. Sci. Ed.).

[9] Wang X., Zhou H., Tong J. (2024). The simplification and analytical verification of the static liquid bridge force model for particle adhesion in inferior seedling substrates. Surf. Interfaces.

[10] Garcia-Gonzalez D., Hack M.A., Kappl M., Butt H.-J., Snoeijer J.H. (2023). Drawing liquid bridges from a thin viscous film. Soft Matter.

[11] Lian G., Thornton C., Adams M.J. (1993). A Theoretical Study of the Liquid Bridge Forces between Two Rigid Spherical Bodies. J. Colloid Interface Sci..

[12] Salem A.E., Wang H., Gao Y., Zha X., Abdeen M.A., Zhang G. (2021). Effect of Biomimetic Surface Geometry, Soil Texture, and Soil Moisture Content on the Drag Force of Soil-Touching Parts. Appl. Sci..

[13] Lu Q., Liu L.J., Liu Z.J., Jin W.T. (2024). Design and experiment of star-tooth spherical disc soil-covering device for planter. Trans. Chin. Soc. Agric. Mach..

[14] Jia H.L., Wang W.J., Wang W.P., Zheng J., Wang Q., Zhuang J. (2018). Application of anti-adhesion structure based on earthworm motion characteristics. Soil Tillage Res..

[15] Jia H.L., Wang W.J., Luo X.F., Zheng J.X., Guo M.Z., Zhuang J. (2016). Effects of profiling elastic press roller on seedbed properties and soybean emergence under double row ridge cultivation. Soil Tillage Res..

[16] Zhang L.M., Zhao W., Wang Z.Y. (2017). The development of the 1ZL-4 type large ridge dense planting ridging and shaping machine. Agric. Mach. Using Maint..

[17] Qian P., He Q., Tang Z., Gu T. (2025). Biomimetic Structural Design for Reducing the Adhesion Between Wet Rice Leaves and Metal Surfaces. Agriculture.

[18] Lin S., Sun H., Yan G., Que K., Xu S., Tang Z., Wang G., Li J. (2025). Structural Design and Analysis of Bionic Shovel Based on the Geometry of Mole Cricket Forefoot. Agriculture.

[19] Torotwa I., Ding Q., Awuah E., He R. (2023). Biomimetic tool design improves tillage efficiency, seedbed quality, and straw incorporation during rototilling in conservation farming. J. Agric. Eng..

[20] Akter A.Y., Basak H. (2022). Design and analysis of biomimetics based excavator bucket and tooth. Proc. Inst. Mech. Eng. Part E J. Process Mech. Eng..

[21] Naziri S., Ridgeway C., Castelo J.A., Ibarra S., Provenghi K., Cortes D.D. (2024). Earthworm-inspired subsurface penetration probe for landed planetary exploration. Acta Geotech..

[22] Guan C., Fu J., Cui Z., Wang S., Gao Q., Yang Y. (2021). Evaluation of the tribological and anti-adhesive properties of different materials coated rotary tillage blades. Soil Tillage Res..

[23] Wang J.W., Wen N., Liu Z.M., Zhou W., Tang H., Wang Q., Wang J. (2022). Coupled bionic design of liquid fertilizer deep application type opener based on sturgeon streamline to enhance opening performance in cold soils of northeast China. Agriculture.

[24] Xu P., Zhang Y., Li L.J., Lin Z., Zhu B., Chen W., Li G., Liu H., Xiao K., Xiong Y. (2022). Adhesion behaviors of water droplets on bioinspired superhydrophobic surfaces. Bioinspir. Biomim..

[25] Zhang Y., Huang H., Liu X., Ren L. (2011). Kinematics of terrestrial locomotion in mole cricket Gryllotalpa orientalis. J. Bionic Eng..

[26] Yang Y., Song X., Li X.J., Chen Z., Zhou C., Zhou Q., Chen Y. (2018). Recent progress in biomimetic additive manufacturing technology: From materials to functional structures. Adv. Mater..

[27] Rapisarda A.C., Dell R. (2025). A kinematic swarm-based approach for simulating stress-strain curves. Math. Mech. Complex Syst..

[28] Pitois O., Moucheront P., Chateau X. (2000). Liquid Bridge between Two Moving Spheres: An Experimental Study of Viscosity Effects. J. Colloid Interface Sci..

[29] Willett C.D., Adams M.J., Johnson S.A., Seville J.P.K. (2000). Capillary Bridges between Two Spherical Bodies. Langmuir.

[30] Rossetti D., Pepin X., Simons S.J.R. (2003). Rupture energy and wetting behavior of pendular liquid bridges in relation to the spherical agglomeration process. J. Colloid Interface Sci..

[31] Wang J.P., Gallo E., François B., Gabrieli F., Lambert P. (2017). Capillary force and rupture of funicular liquid bridges between three spherical bodies. Powder Technol..

[32] Xu P.F., Duan S.Y., Wang F. (2020). Reverse modeling and topological optimization for lightweight design of automobile wheel hubs with hollow ribs. Int. J. Comput. Methods.

[33] Wu S., Liu C., Sun H., Hu J., Li Y., Guo W. (2025). Numerical Simulation and Orthogonal Test of Droplet Impact on Soybean Leaves Based on VOF Method and High-Speed Camera Technology. Agronomy.

[34] Li J., Qi H., Ma Y., Gao P., Wu B. (2024). Simulation and Structural Analysis of a Flexible Coupling Bionic Desorption Mechanism Based on the Engineering Discrete Element Method. Biomimetics.

[35] Liu S., Wang Y., Jiang H., Wei Q., Xiao J. (2016). Aerial survey and scanning point cloud data acquisition & modeling technology for hydropower engineering. Proceedings of the 2016 5th International Conference on Advanced Materials and Computer Science.

[36] Ucgul M. (2023). Simulating soil–disc plough interaction using discrete element method–multi-body dynamic coupling. Agriculture.

[37] Mei X., Du H., Yao W., Liu A. (2025). Investigating the Wear Evolution and Shape Optimize of SAG Mill Liners by DEM-FEM Coupled Simulation. Minerals.

[38] Li J.W., Jiang X.H., Ma Y.H., Tong J., Hu B. (2020). Bionic Design of a Potato Digging Shovel with Drag Reduction Based on the Discrete Element Method (DEM) in Clay Soil. Appl. Sci..

[39] Zhong G.Y., Li H.W., He J., Wang Q., Lu C., Wang C., Tong Z., Cui D., He D. (2023). Design and test of single-disc opener for no-till planter based on support cutting. Agriculture.

[40] Liu G., Han X., Wang Z., Wang K., Zhang Z., Duan Z. (2024). Viscosity Reduction and Drag Reduction Performance Analysis of Bionic Excavator Buckets Based on Discrete Element Method. Biomimetics.

[41] Wu B., Zhang R., Hou P., Tong J., Zhou D., Yu H., Zhang Q., Zhang J., Xin Y. (2021). Bionic Nonsmooth Drag Reduction Mathematical Model Construction and Subsoiling Verification. Appl. Bionics Biomech..

[42] Sun Y., Wang Y., Wang L. (2023). Digging Performance and Stress Characteristic of the Excavator Bucket. Appl. Sci..

[43] Cuomo M., Contrafatto L., Greco L. (2024). A cohesive interface model with degrading friction coefficient. Math. Mech. Complex Syst..

